# Measuring childhood mortality through mobile phone interviews in Mozambique

**DOI:** 10.1111/tmi.70004

**Published:** 2025-09-01

**Authors:** Almamy Malick Kante, Cremildo Manhica, Akum Aveika, Azarias Mulungo, Fred Van Dyk, Nordino Machava, Helen Kuo, Charfudin Saccor, Dustin G. Gibson, Celso Monjane, Robert E. Black, Ivalda Macicame, Agbessi Amouzou

**Affiliations:** ^1^ Department of International Health Johns Hopkins Bloomberg School of Public Health Baltimore Maryland USA; ^2^ Instituto Nacional de Saúde Maputo Mozambique; ^3^ Centro de Investigação em Saude de Manhiça (CISM) Maputo Mozambique; ^4^ Facultat de Medicina i Ciències de la Salut Universitat de Barcelona (UB) Barcelona Spain

**Keywords:** childhood mortality, countrywide mortality surveillance for action, full pregnancy history, mobile phone surveys, Mozambique, random digit dialling

## Abstract

**Objectives:**

Childhood mortality is a key indicator of progress in health and development in low‐ and middle‐income countries, traditionally measured through household surveys with face‐to‐face interviews. This study explored an alternative approach that used mobile phone interviews with women in Mozambique.

**Methods:**

Using two sampling approaches, we interviewed women of reproductive age about their pregnancy history through mobile phones. The first method used an existing database of phone numbers collected from a national mortality surveillance, Countrywide Mortality Surveillance for Action (COMSA). The second employed random digit dialling (RDD) to generate phone numbers. The COMSA phone sample successfully reached 13,545 women while the RDD sample reached 10,359 women. We compared neonatal (NMR), infant (IMR) and under‐five mortality rates (U5MR) to estimates from the United Nations (UN), COMSA and the 2022 Demographic and Health Survey (DHS). The mobile phone‐based mortality rates were adjusted using the raking approach.

**Results:**

The mobile phone interviews incorporating pregnancy history yielded recent childhood mortality rates comparable to those reported by the DHS. The 2020–2021 U5MRs were estimated at 59.3 (95% confidence interval [95% CI]: 41.9–76.7) in the COMSA phone sample and 44.9 (95% CI: 9.0–80.7) in the RDD sample, compared to 59.6 (95% CI: 53.7–65.6) in the DHS. These estimates were lower than the UN projections at 71.6 (95% CI: 65.5–87.1) and COMSA at 80.0 (95% CI: 69.0–91.0). We observed similar trends for NMR and IMR. Childhood mortality trends were comparable between the COMSA phone sample and the DHS sample. In contrast, the RDD sample appeared to consistently underestimate childhood mortality compared to the other samples.

**Conclusion:**

Mobile phone surveys, including standard full pregnancy history tools, produced recent childhood mortality levels and trends for national and subnational levels similar to face‐to‐face approaches such as the DHS.

## INTRODUCTION

Childhood mortality rates are important indicators for monitoring progress in health and development, particularly in low‐ and middle‐income countries (LMIC), which require frequent measurements [1]. The current standard for directly measuring childhood mortality in household surveys in LMIC involves face‐to‐face interviews with women aged 15–49 about their birth or pregnancy histories [2]. These surveys gather data on births, including miscarriages, stillbirths and child deaths [3, 4]. The detailed, complex and sensitive nature of the questionnaire has been thought to require face‐to‐face interviews that allow probing and jogging the respondents' memory to ensure accurate capture of information on dates of events and ages of the deaths. The Demographic and Health Survey (DHS) program initially used full birth histories but recently transitioned to full pregnancy histories following further evidence of the superiority of this approach [3]. Data enable trend analysis in mortality among children under age 5 going back to 15 years preceding the survey [5, 6, 7] and also capture mortality in older children aged 5–14 years [5, 8]. The estimates can be broken down by socio‐economic and demographic characteristics and other relevant characteristics collected in the survey [2].

The COVID‐19 pandemic and resulting lockdowns in 2020–2021 exposed the limitations of approaches solely relying on face‐to‐face interviews. The inability to conduct such interviews led to a significant data gap affecting global and country monitoring activities. Mobile phone interviews for obtaining similar mortality measures may offer a viable alternative, providing quicker and cost‐effective mortality data collection without face‐to‐face interviews [9, 10]. While mobile interviews were successfully conducted before the pandemic [9, 11, 12, 13, 14, 15, 16], they have not been used for childhood mortality in large populations or at the national level using standard, rigorous tools such as full birth or pregnancy histories. With increasing mobile phone access and literacy [17], this approach could potentially reach a representative population sample. It can measure excess mortality during pandemics or crises and complement continuous population surveillance.

Data collection using mobile phone interviews can take various forms, including direct phone calls, short message service‐based approaches and interactive voice response (IVR) [11, 12]. The sampling frame is often derived from an existing list of phone numbers. However, strategies relying on random digit dialling (RDD) using computer‐generated random phone numbers have also been tested [13, 18, 19]. These different strategies yield varying population samples or response rates [15, 18, 20]. While mobile phone surveys can be low cost [15, 20, 21], their usual very low response rates and reaching only individuals who own mobile phones create further challenges in obtaining representative population estimates [14]. Researchers have employed sample quota monitoring and post‐weighting adjustments to correct the estimates [19, 22, 23]. Therefore, mortality estimates from mobile phone interviews must be carefully assessed for accuracy. Nonetheless, this approach can be a valuable tool in hard‐to‐reach settings or during pandemics when timely mortality and disease data are essential, and face‐to‐face interviews are not feasible. Our study explored this approach by incorporating pregnancy histories to measure childhood mortality.

This paper reports a national mobile phone childhood mortality assessment conducted in Mozambique, as part of the Rapid Mortality Mobile Phone Survey (RaMMPS) project.

## METHODS

### Study design, procedures and data

RaMMPS is an innovative approach that utilises mobile phone interviews to collect standard tools from DHS to estimate mortality across five LMICs: Bangladesh, Burkina Faso, the Democratic Republic of Congo, Malawi and Mozambique. In Mozambique, the project aimed to collect birth and pregnancy history data to estimate national and subnational (provincial and residence area) childhood mortality rates. The initiative was jointly implemented by the Mozambique National Institute of Health and Johns Hopkins University [24]. Mozambique is a sub‐Saharan African country with a population of about 32 million in 2022, residing in 6 million households [25]. Approximately 70% of the population comprised adults aged 15 years and above. The country's mobile network is dominated by three operators (Tmcel, Vodacom and Movitel), with Movitel and Vodacom leading the coverage. Mobile phone penetration grew by 26% from 2017 to 2021, reaching almost 14 million active subscriptions [26]. Mobile phone owners often subscribe to two or more companies [26]. About 69% of households had mobile phone access in 2022, varying by urban (86%) and rural (60%) areas [27]. However, the mobile cellular subscription rate was 42 per 100 people in Mozambique in 2022, among the lowest in the world [17].

RaMMPS study used two sampling approaches. The first utilised the existing database of phone numbers from the ongoing Countrywide Mortality Surveillance for Action in Mozambique (COMSA) [28], a nationally representative mortality and cause of death surveillance system covering about 850,000 population across all provinces. A province‐stratified random sample of 48,271 households was drawn to successfully reach 15,000 women of reproductive age.

The second approach used RDD to generate a nationwide sampling frame. Based on Mozambique's phone number structure, we randomly generated numbers for all three mobile network operators using a statistical code [13, 23]. Calls were made via a third‐party web‐based platform capable of handling large volumes [29]. A pre‐test revealed that 13.7% (1784/12,968) of phone numbers were picked up, and only 2.6% (46/1784) were eligible and willing to participate. We developed an IVR system to screen participants for potential eligibility. The IVR collected information on the respondent's language and interest in participating in the study. A live trained interviewer followed up with interested respondents to conduct the interview using a Computer‐Assisted Telephone Interview. Although we targeted women of reproductive age for the survey, all respondents expressing interest were followed up, including men, where we asked to speak to their spouse or an eligible female in the household. To reach a representative sample in each province, we developed a quota monitoring sample using the distribution for age groups (15–19, 20–29, 30–39 and 40–49 years) and urban–rural residence [19] based on the 2017 population census [30].

Candidates for data collection were tested for language proficiency in both Portuguese and their stated local languages. Selected candidates were trained for 10 days, covering data collection protocols, ethics and good practices, Open data kit (ODK) [31] application and pilot tools. A total of 32 interviewers and 4 supervisors were selected, with women comprising 89%. A call centre was set up and equipped with the necessary tools to facilitate clear communication between interviewers and respondents. Additionally, Wi‐Fi connectivity was established to support access to the *CallTrack* application and ODK server for daily data transfer, storage and backup. Following the pilot, the main surveys data collection was implemented successively, starting with the COMSA phone sample between March and August 2022, followed by the RDD/IVR sample from June to December 2022.

The COMSA phone sample used a full pregnancy history module, while the RDD/IVR sample was randomised to a full pregnancy history and a seven‐year truncated pregnancy history. The randomisation aimed to compare the full and the truncated pregnancy histories in accurately generating mortality estimates. We achieved equal samples during data collection for each arm.

A total of 48,271 phone numbers were contacted with 106,738 calls made, successfully interviewing 13,247 women aged 15–49 in the COMSA phone sample. In the full pregnancy history arm of the RDD/IVR sample, 22,786 phone numbers were contacted with 40,964 calls made, successfully interviewing 5059 women aged 15–49. Approximately 45% of phone numbers were reached on the first attempt, with 40.2% in the COMSA phone sample and 51.2% in the RDD/IVR sample. Data collectors were trained to make at least six call attempts, but less than 2% of phone numbers required the full six attempts.

### Analysis

Disposition codes for each phone call were assigned per American Association for Public Opinion Research standards [32]. We calculated the survey response and refusal rates using formulas of Response Rate #1 (RR1) and Refusal Rate #1 (REF1), that is, by dividing the number of completed interviews or refusals by the total number of respondents who either met the inclusion criteria or whose eligibility could not be determined [32]. This approach allowed us to assess participation, including those whose inclusion status was uncertain, ensuring a comprehensive evaluation of the response and refusal metrics in the study. We restricted the analysis of survey completion times to interviews lasting 1–60 min to exclude unrealistic values. We then calculated the average time to administer the full pregnancy history instrument, including summary pregnancy history questions.

We focused on assessing national and subnational (provincial and place of residence) level childhood mortality using data from the COMSA phone sample and the full pregnancy history from the RDD/IVR sample. To understand any systematic distortion in the samples, we first compared the distribution of the sample of women in both surveys to the 2017 Population Census data based on place of residence, age group, education and province. Using the DHS as the reference data set [2], we assessed the quality of the mortality data by analysing annual trends in the number of births and deaths over the past 10 years. Additionally, we examined the distribution of age at death, the proportion of deaths of 0–1 and 0–6 days out of all neonatal deaths and the proportion of neonatal deaths out of all under‐five deaths. Further, we assessed the average number of children ever born and children who died by women's age group, comparing these findings with data from the 2022 Mozambique DHS [27].

We computed mortality rates—neonatal (NMR), infant (IMR) and under‐five (U5MR) for 2‐year periods—from 2012 to 2021. The trend was then compared to the national trend estimated by the United National Interagency Group for Mortality Estimation (UN‐IGME) [5]. We also computed mortality rates for 5‐year periods for national and subnational level estimates and compared them with the 2022 DHS estimates [27], the main COMSA study estimates [28] and the 2017 Mozambique population census projections for 2018–2021. We computed standard errors of the estimates using the jackknife method [33, 34]. The mortality estimates from the RaMMPS, the main COMSA and the 2022 DHS are reported with a 95% confidence interval (95% CI), while the UN‐IGME estimates are reported with a 90% CI. Mortality estimates derived from mobile phone interviews reflect only rates among phone owners, who generally have a higher socio‐economic status than non‐owners [35]. Consequently, these rates are likely lower than representative estimates. To address this, we applied post‐adjustment weighting using the raking approach [35, 36]. Distributional estimates for the raking were based on women's age group (15–19, 20–29, 30–39, or 40–49), place of residence (urban or rural), educational attainment (none, primary, secondary or higher level) and household size (less than 5, 5–7 or 8 or more) [30], using the STATA package “svycal” [37]. All analyses used STATA statistical package version 17 [38].

Ethical clearance for the study was obtained from the Ethics Committee of Johns Hopkins Bloomberg School of Public Health (#16261) and the Institutional Committee for Bioethics in Health of Mozambique (#35/CIBS‐INS/2021). The study adhered to the relevant local laws and the guidelines the institution overseeing the research set. We obtained informed consent from all participants before the data collection began. Consent forms were translated into Portuguese and the local languages spoken in the respective provinces. Only emancipated minors, defined as married minors aged 15–17 years old, were included and provided oral consent. Adults who lacked the capacity to consent were excluded from the study. We requested a waiver for written consent since the study was conducted via cellphone, a measure taken to protect data collectors and participants from COVID‐19 transmission risks.

## RESULTS

### Sample distribution

Table 1 compares the raw distribution of women 15–49 years old from the COMSA phone sample (*n* = 13,247) and the RDD/IVR sample (*n* = 5059) with the distribution from the 2022 Mozambique DHS (*n* = 13,183) by selected characteristics. Overall, these raw distributions are not consistent with the 2022 DHS. The mobile phone samples were overly represented in urbanised provinces such as Maputo City and Maputo Province, while the most populous provinces according to the 2022 DHS were Nampula and Zambezia, with 23% and 17% of the women 15–49 years old population, respectively. Urban residents were overly represented in the mobile phone samples, with 60% for the COMSA and over 80% for RDD/IVR. Both mobile phone samples severely under‐reached adolescents aged 15–19 and over‐reached older women aged 30 years or more, especially in the COMSA phone sample. Regarding formal education, women of secondary or more were more represented in mobile phone samples than those with no or primary education. We observed mixed marital status and parity patterns in both mobile phone samples.

**TABLE 1:** Distribution of samples by selected characteristics of women aged 15–49.

Characteristics	Categories	COMSA phone (*n* = 13,247)				RDD/IVR (*n* = 5059)				2022 DHS (*n* = 13,183)			
		Unweighted		Weighted		Unweighted		Weighted		Unweighted		Weighted	
		*n*	%	*n*	%	*n*	%	*n*	%	*n*	%	*n*	%
Age group	15–19	686	5.2	3028	22.9	584	11.5	1044	20.6	3109	23.6	3050	23.1
	20–29	4655	35.1	4996	37.7	2311	45.7	1904	37.6	4695	35.6	4888	37.1
	30–39	4261	32.2	3073	23.2	1425	28.2	1186	23.5	3121	23.7	3063	23.2
	40–49	3645	27.5	2150	16.2	740	14.6	926	18.3	2258	17.1	2182	16.6
Education	None	1421	10.7	2977	22.5	185	3.7	1101	21.8	3033	23.0	3522	26.7
	Primary	4895	37.0	6591	49.8	1234	24.4	2503	49.5	5426	41.2	5601	42.5
	Secondary	6931	52.3	3679	27.8	3641	72.0	1456	28.8	4724	35.8	4060	30.8
Marital status	Never been married	2276	17.2	2108	15.9	1401	27.7	1155	22.8	8195	62.2	8488	13.7
	Married	9151	69.1	9892	74.7	3158	62.4	3394	67.1	1853	14.1	1799	64.4
	Widowed/separated/divorced	1820	13.7	1247	9.4	501	9.9	511	10.1	3135	23.8	2896	22.0
Parity	0	1804	13.6	2468	18.6	1219	24.1	1210	23.9	3227	24.5	3065	23.3
	1–3	7118	53.7	6766	51.1	2939	58.1	2508	49.6	5920	44.9	6028	45.7
	4 and more	4325	32.7	4013	30.3	902	17.8	1342	26.5	4036	30.6	4089	31.0
Province	Niassa	607	4.6	811	6.1	136	2.7	298	5.9	1113	8.4	861	6.5
	Cabo Delgado	821	6.2	1046	7.9	183	3.6	430	8.5	1314	10.0	705	5.4
	Nampula	607	4.6	2606	19.7	465	9.2	857	16.9	1446	11.0	3064	23.3
	Zambezia	549	4.1	2384	18.0	419	8.3	1013	20.0	976	7.4	2193	16.6
	Tete	700	5.3	1248	9.4	262	5.2	455	9.0	1168	8.9	1314	10.0
	Manica	840	6.3	923	7.0	306	6.1	353	7.0	1196	9.1	909	6.9
	Sofala	1193	9.0	1107	8.4	506	10.0	454	9.0	1218	9.2	909	6.9
	Inhambane	1460	11.0	708	5.3	416	8.2	257	5.1	1008	7.7	555	4.2
	Gaza	1007	7.6	675	5.1	370	7.3	241	4.8	1276	9.2	670	5.1
	Maputo Province	2333	17.6	1161	8.8	1131	22.4	473	9.4	1276	9.7	1347	10.2
	Maputo City	3130	23.6	578	4.4	866	17.1	229	4.5	1259	9.6	655	5.0
Residence area	Urban	8176	61.7	5075	38.3	4244	83.9	1993	39.4	5695	43.2	5120	38.8
	Rural	5071	38.3	8172	61.7	816	16.1	3067	60.6	7488	56.8	8063	61.2
Total		13,247	100.0	13,247	100.0	5060	100.0	5060	100.0	13183.0	100.0	13183.0	100.0

Abbreviations: COMSA phone, Countrywide Mortality Surveillance for Action phone sample; RDD/IVR, random digit dialling/interactive voice response sample; 2022 DHS, 2022 Mozambique Demographic and Health Survey.

### Survey response rate and duration

The response and refusal rates (RR1 and REF1) were 35.8% and 7.6% for the COMSA phone sample and 32.3% and 7.2% for the RDD/IVR sample. The median duration to administer the full pregnancy history module was 4.37 [Q1–Q3: 2.18–6.55] for the COMSA phone sample and 2.18 min [Q1–Q3: 2.18–4.37] for the RDD/IVR sample.

### Quality of mortality data

#### Distribution of births and under‐five deaths

Figure A1 compares the birth year distribution of births from COMSA phone and RDD/IVR samples to 2022 Mozambique DHS (2012–2021). The annual number of births from the COMSA phone sample was slightly above 1500 between 2012 and 2019 and dropped slightly below 1500 in 2019 and 2020. In contrast, the distribution of births in the RDD/IVR sample shows a steady increase from 2012 to 2021, aligning closely with the birth trends from 2022 DHS.

Figure A2 compares the birth year distribution of under‐five deaths from COMSA phone and RDD/IVR samples to 2022 Mozambique DHS (2012–2021). The patterns differ across the RaMMPS samples, with the COMSA phone sample showing more similar patterns to the 2022 DHS, except that there was an increase in 2021. However, the RDD/IVR showed more irregular patterns.

Figures A3, A4, and A5 compare the neonatal and under‐five death patterns from COMSA phone and RDD/IVR samples to 2022 Mozambique DHS (2012–2021). The patterns differ across the RaMMPS samples, with the RDD/IVR showing more irregular patterns. The COMSA phone sample aligns with the 2022 DHS trends, showing three‐quarters of neonatal deaths occurring within the first week (Figure A4) and half occurring during the first 2 days (0–1) (Figures A3). The RDD/IVR sample has severe under‐reporting of early neonatal deaths in 2017 (with no deaths on days 0–1) and over‐reporting in earlier years (2012–2013) or later years (2019–2021) (Figures A3). The proportion of neonatal deaths among under‐five deaths in the COMSA phone sample increased steadily from 30% in 2012 to 55% in 2021. However, the pattern was irregular in the RDD/IVR sample, with substantial under‐reporting in 2015 and 2017 and possible over‐reporting in 2018 (Figures A5).

#### Average number of children by woman's age group

Figures A6 and A7 compare the average number of children ever born and children who died by mother's age group in the COMSA phone sample, RDD/IVR sample and the 2022 Mozambique DHS. As expected, all three sources show similar patterns with an increase in parity with age, though at varying levels (Figure A6). The RaMMPS samples generally report lower averages than the 2022 DHS, with the RDD/IVR sample consistently underestimating both the number of children ever born and child mortality compared to the COMSA phone sample.

### National childhood mortality trends—2012–2021

**FIGURE 1:**
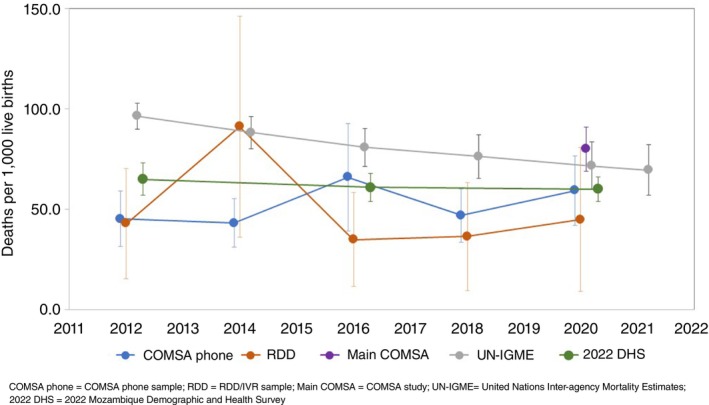
Trends in under‐five mortality rates by samples across different sources, 2012–2021 (2‐year period).

Figure 1 compares U5MR trends and levels across five sources from 2012 to 2021. The COMSA phone and RDD/IVR samples used two‐year rates computed retrospectively from the survey year. These are compared with 5‐year rates from the 2022 DHS, the yearly UN‐IGME estimates and the 2019–2020 main COMSA estimates. U5MR estimates from the COMSA phone sample (59.0 per 1000 [95% CI: 45.6–72.4]) in 2020 aligned with the DHS and were slightly higher than the RDD/IVR sample (44.9 [95% CI: 9.0–80.7]). However, those estimates were lower than the UN‐IGME and main COMSA but had overlapping confidence intervals. The RDD/IVR sample generally reported lower U5MRs than the COMSA phone sample, except in 2014–2015. While comparable to the DHS estimates, U5MR from the COMSA phone sample did capture similar trends as the UN‐IGME (as was the 2022 DHS). However, the U5MR trend from the RDD/IVR sample was different from other sources.

Table 2 compares NMR and IMR trends and levels across five sources from 2012 to 2021. For IMR, estimates from the population census projections in 2018–2021 were included. All sources appear to have comparable estimates of NMR in the recent period (the year 2020), ranging from 22 (COMSA phone sample) to 28 per 1000 (UN‐IGME). While the COMSA phone sample and DHS captured similar NMR trends, the RDD/IVR sample displayed more irregular patterns. All empirical sources showed stagnating NMR, contrasting with the declining trends in the modelled estimates from UN‐IGME. A similar discrepancy was observed for IMR. Both RaMMPS samples were aligned with the DHS estimates in 2020 (ranging from 34 to 39 per 1000) but were lower than the UN‐IGME (52 per 1000), COMSA (46 per 1000) and the population census projections (67 per 1000). The COMSA phone sample captured a similar flat IMR trend as the DHS, different from the higher levels and decreasing trends estimated by UN‐IGME.

**TABLE 2:** Trends in neonatal and infant mortality rates across different sources across different sources, 2012–2021 (2‐year period).

Indicator	Year/period	COMSA phone		RDD/IVR		COMSA		2022 DHS		Census	UN‐IGME	
		Estimates	95% CI	Estimates	95% CI	Estimates	95% CI	Estimates	95% CI	Estimates	Estimates	90% CI
Infant mortality rates	2012	26.6	[16.2–36.9]	22.4	[2.2–42.5]			40.0	[34.0–45.0]		65.4	[58.9–71.9]
	2014	29.5	[20.7–38.4]	78.6	[24.2–133.1]						61.9	[53.9–69.9]
	2016	51.7	[25.3–78.1]	16.8	[3.9–29.7]			39.0	[34.0–45.0]		58.9	[49.4–68.3]
	2018	35.8	[24.3–47.3]	27.1	[4.5–49.8]					69.7	55.2	[44.4–66.0]
	2020	34.1	[20.2–47.9]	35.6	[0.2–71.0]	46	[38.1–53.9]	38.9	[34.0–44.0]	67.4	52.5	[40.5–64.5]
Neonatal mortality rates	2012	7.9	[1.8–14.0]	6.6	[−0.8–13.9]			18.0	[14.0–22.0]		32.9	[24.7–41.2]
	2014	20.0	[12.0–27.9]	42.9	[10.5–75.3]						31.8	[23.2–40.5]
	2016	22.4	[1.5–43.4]	10.6	[−1.6–22.7]			19.0	[15.0–23.0]		30.5	[21.2–39.9]
	2018	20.5	[12.5–28.5]	21.6	[−0.7–43.8]						29.4	[19.3–39.5]
	2020	22.9	[10.3–35.5]	26.4	[−8.6–61.3]	23.2	[18.2–28.2]	24.3	[20.0–29.0]		28.1	[17.5–38.6]

Abbreviations: Census, Mozambique 2017 Housing and Population Census Mortality projections; COMSA, Countrywide Mortality Surveillance for Action main study; COMSA phone, Countrywide Mortality Surveillance for Action phone sample; RDD/IVR, random digit dialling/interactive voice response sample; UN‐IGME, United Nations Inter‐agency Mortality Estimates; 2022 DHS, 2022 Mozambique Demographic and Health Survey.

### National childhood mortality rates—2017–2021

We also analysed five‐year NMR, IMR, and U5MR from both RaMMPS samples and compared them to the 2022 Mozambique DHS. Table 3 compares NMR, IMR, and U5MR estimates across three sources at national and residence area levels from 2017 to 2021. Mortality rates in the COMSA phone sample on 2017–2021 aligned with the 2022 DHS (U5MRs: 59.0 [95% CI: 45.6–72.4]; IMR: 41.0 [95% CI: 28.8–53.2] and NMR: 22.6 [95% CI: 13.3–32.0]). Those estimates were higher than the RDD/IVR sample (U5MR: 40.4 [95% CI: 22.0–58.8]; IMR: 29.6 [95% CI: 13.2–46.0] and NMR: 21.8 [95% CI: 5.7–37.9]).

**TABLE 3:** Neonatal, infant and under‐5 mortality rates across different sources at national and residence area levels, 2017–2021.

Level	Indicator	COMSA phone		RDD/IVR		2022 DHS	
		Estimates	95% CI	Estimates	95% CI	Estimates	95% CI
National	NMR	22.6	[13.3–32.0]	21.8	[5.7–37.9]	24.0	[20.0–28.0]
	IMR	41.0	[28.8–53.2]	29.6	[13.2–46.0]	39.0	[34.0–44.0]
	U5MR	59.0	[45.6–72.4]	40.4	[22.0–58.8]	60.0	[54.0–66.0]
Urban	NMR	20.4	[11.5–36.5]	12.7	[6.1–53.0]	24.3	[17.5–29.8]
	IMR	34.7	[21.2–60.4]	24.8	[15.5–58.5]	36.8	[28.3–46.2]
	U5MR	49.1	[33.1–81.3]	33.6	[21.2–73.4]	50.2	[40.3–70.6]
Rural	NMR	23.6	[10.8–48.0]	27.4	[1.7–59.1]	24.3	[18.8–47.3]
	IMR	43.8	[27.3–81.6]	32.5	[6.5–74.1]	39.7	[33.2–74.5]
	U5MR	63.3	[45.4–114.3]	44.6	[15.8–94.6]	63.4	[56.2–110.9]

Abbreviations: COMSA phone, Countrywide Mortality Surveillance for Action phone sample; IMR, infant mortality rates; NMR, neonatal morality rates; U5MR, under‐five mortality rates; RDD/IVR, random digit dialling/interactive voice response sample; 2022 DHS = 2022 Mozambique Demographic and Health Survey.

### Subnational childhood mortality rates—2017–2021

U5MR from both RaMMPS samples varied by urban and rural residences as expected, although differences were not statistically significant (Table 3). U5MRs from the COMSA phone sample were similar to those from the 2022 DHS (approximately 63 per 1000 in rural residences and 50 per 1000 in urban residences). NMRs were similar in rural residences in both samples (about 24 per 1000) but lower in urban residences in the COMSA phone sample (20.4 [95% CI: 11.5–29.3]) compared to the DHS (24.3 [95% CI: 17.5–31.1]). U5MRs were higher in the COMSA phone sample than the RDD/IVR sample across urban and rural residences. In contrast, NMR was higher in the RDD/IVR sample. Urban NMRs were estimated at 20.4 [95% CI: 11.5–29.3] for the COMSA phone sample and 12.7 [95% CI: 6.1–19.3] for the RDD/IVR sample. However, rural NMRs were estimated at 23.6 [95% CI: 10.8–36.5] for the COMSA phone sample and 27.4 [95% CI: 1.7–53.0] for the RDD/IVR sample. We observed large variations at the provincial level in both samples, with the RDD/IVR sample generally underestimating mortality rates compared to the COMSA phone sample. However, mortality estimates were unreliable for many provinces in the RDD/IVR sample particularly (Figure A7).

### DISCUSSION

The recent expansion of mobile phone networks and phone ownership in LMICs offers an increased opportunity to explore approaches for remote data collection through mobile phone interviews. The RaMMPS project is among the first to test the feasibility and accuracy of using standard mortality measurement tools in mobile phone surveys to estimate childhood mortality levels and trends. This study tested two distinct strategies, one relying on a sample of phone numbers drawn from a nationally representative mortality surveillance platform in Mozambique, known as COMSA [28] and another utilising RDD with pre‐screening through IVR. While previous studies have employed mobile phone interview strategies in LMICs [9, 11, 12, 13, 14, 19, 35], none have attempted to implement a full pregnancy history module in a large and nationally drawn sample of women of reproductive age. The complexity and sensitivity involved in collecting full pregnancy history information make this approach especially challenging, highlighting the need to test the reliability of the mobile phone interview as an approach for rapid child mortality monitoring.

A total of 13,247 women aged 15–49 years were interviewed in the COMSA phone sample and 5059 women were interviewed in the RDD/IVR sample. The study demonstrated that mortality data can be collected through mobile phone interviews using standard mortality measurement tools such as the full pregnancy history module. The survey achieved response rates of 35.8% in the COMSA phone sample and 32.3% in the RDD/IVR sample, aligning closely with a similar study in Burkina Faso (33%) [39] and surpassing another similar study in Malawi (26.6%) [40]. The refusal rates in both RaMMPS samples were also quite low, approximately 6%, matching those in Burkina Faso (6%) [39], but lower than those observed in the Malawi study (10.4%) [40]. The average time to complete the full pregnancy history module was 4.4 min, slightly longer than the Malawi study (3.2 min) [40] but twice as fast as a multi‐country study with face‐to‐face interviews conducted in Guinea‐Bissau, Ethiopia, Uganda, Bangladesh and Ghana [3]. Shorter interview times in mobile phone surveys might be related to various factors, including under‐sampling of key demographic groups with higher childhood mortality and fertility rates, such as less educated or rural women [27, 41], typically harder to reach through mobile surveys [10, 14]. Despite these limitations, mobile phone surveys, with shorter completion times and moderate response rates, offer a promising alternative for collecting mortality data, especially in challenging contexts such as during public health emergencies.

The findings highlight the strong potential of mobile phone‐based approaches in measuring recent childhood mortality levels, particularly when compared to current standard practices such as the DHS that typically collect child mortality data using full pregnancy history of women aged 15–49 years [27]. In 2020, the U5MR from the COMSA phone sample (59.0 [95% CI: 45.6–72.4]) was higher than from the RDD/IVR sample (44.9 [95% CI: 22.0–58.8]), but closely aligned with the 2022 DHS estimates [27], reinforcing the validity of mobile‐based data collection methods. NMR and IMR across the RaMMPS samples followed similar patterns as U5MR. The COMSA phone sample showed childhood mortality trends consistent with the 2022 DHS findings [27]. However, the RDD/IVR sample substantially underestimated childhood mortality and did not produce consistent trends. Several factors may explain these discrepancies. First, the RDD/IVR sample had a higher proportion of urban and younger women than the COMSA phone sample. Second, the RDD/IVR sample was carried out concurrently with a truncated pregnancy history module implemented by the same data collectors using a randomised approach, potentially affecting the data accuracy. Third, the randomisation between the full pregnancy history versus the truncated pregnancy history reduced the sample size to about half of the COMSA phone sample. Finally, mobile phone‐based childhood mortality rates were consistently lower than UN‐IGME estimates. However, this is not entirely surprising given that the UN‐IGME estimates are based on statistical curve fitting and incorporate historical trends that projected past declines in mortality rates [5, 28, 42].

We also found variations in U5MRs by place of residence across the RaMMPS samples, with higher mortality rates in rural areas than in urban areas. U5MR was lower in the RDD/IVR sample than the COMSA phone sample, with the latter closely aligning with the 2022 DHS [27], reinforcing the reliability of mobile‐based surveys for tracking subnational mortality rates. However, we observed large variations at the provincial level, with nearly half of the provinces showing unreliable estimates in the RDD/IVR sample, likely due to the low completion rates in provinces such as Niassa, Cabo Delgado, Tete and Inhambane. To enhance representativeness, we developed a quota monitoring sample using the distribution for age groups (15–19, 20–29, 30–39 and 40–49 years) and urban–rural residence. However, less than 60% of the target sample was reached in those provinces. To address potential selection bias, we applied post‐adjustment weights, accounting for age group, place of residence, educational attainment and household size, to ensure that our sample accurately reflects the population of Mozambique. However, this approach seems to accurately adjust the probable missing data in the RDD/IVR sample. While various post‐survey adjustment approaches have been proposed for mobile phone survey data in LMICs, particularly in health studies [9, 10, 11, 15, 16, 18, 20, 22, 23], further research is needed specifically on childhood mortality indicators [35, 39].

We found little differences in data quality between the COMSA phone sample and the 2022 DHS. This assessment was based on yearly birth and death trends, the distribution of under‐five deaths by age group and the average number of children born and died by woman's age group. However, the RDD/IVR sample showed an irregular pattern in the proportion of neonatal deaths among under‐5 deaths, with substantial under‐reporting compared to COMSA phone and 2022 DHS samples. This suggests possible missing deaths in the RDD/IVR data, raising concerns about the accuracy of child death distribution across age groups and periods. The discrepancies may be attributed to various factors, such as the over‐representation of urban and highly educated women in the RDD/IVR sample. Furthermore, three‐quarters of neonatal deaths occurred within the first week of life, with half occurring within the first 2 days. The proportion of under‐five deaths classified as neonatal (about one third) was consistent across the RaMMPS samples, the 2022 DHS and the UN estimates over the last decade [5, 28].

### Limitations

This study faced several limitations, primarily due to the relatively small sample size of the RDD/IVR sample, which may have impacted the estimates and reduced the robustness of the conclusions. Compared to the COMSA phone sample, these limitations highlight the need for further research to enhance the reliability and validity of findings. One significant challenge was reaching the target sample, particularly in Mozambique's rural and densely populated provinces, where two thirds of the population resides. In 2022, Mozambique ranked among the lowest seven countries in mobile cellular subscriptions, with 42 subscriptions per 100 people [17]. However, about two thirds of households had access to a mobile phone, with large differences between urban (86%) and rural residences (60%) [27]. Therefore, the mobile phone samples were disproportionally represented in urbanised areas, such as Maputo City and Maputo Province. Further, the study showed low participation among younger women (under 20 years old) and those without formal education, contributing to sample bias. This underrepresentation highlights the need for careful post‐adjustment of the survey weights to improve the sample's representativeness at both national and subnational levels. Consequently, the small sample size limits the study's ability to draw definitive conclusions about childhood mortality trends or produce reliable provincial‐level estimates. The skewed representation of certain demographic groups and the limited number of respondents introduced challenges in generalising mortality patterns across the broader population.

## CONCLUSION

This study is among the first to use mobile phone interviews to collect standard full pregnancy history data. Our approach produced recent childhood mortality levels and trends for national and subnational levels comparable to those obtained through similar face‐to‐face approaches such as the DHS. This approach is particularly valuable in settings where face‐to‐face surveys are challenging, such as during health crises (e.g., Ebola and Cholera outbreaks, COVID‐19 pandemic) or humanitarian emergencies (e.g., armed conflicts, displacement crises, natural disasters). While the findings provide important preliminary insights, they underscore the need for further research. The post‐survey weighting adjustments help address some challenges, but larger sample sizes and more comprehensive data collection methods are essential to improve outreach to underrepresented groups. Expanding these efforts will be critical to establishing clearer, more reliable patterns in childhood mortality across different provinces or demographic groups.

## FUNDING INFORMATION

This study was made possible with financial support from the Bill and Melinda Gates Foundation (INV‐023211). The funder had no role in study design, data collection, data analysis, data interpretation, or report writing.

## CONFLICT OF INTEREST STATEMENT

The authors declare no conflicts of interest.

## Supporting information


**Data S1.** Supporting Information.
