# Supplementary figures and images for: Measuring childhood mortality through mobile phone interviews in Mozambique
Source: Trop Med Int Health. 2025 Sep 1;30(10):1087–96. doi: 10.1111/tmi.70004 (PMC12501560; doi:10.1111/tmi.70004)

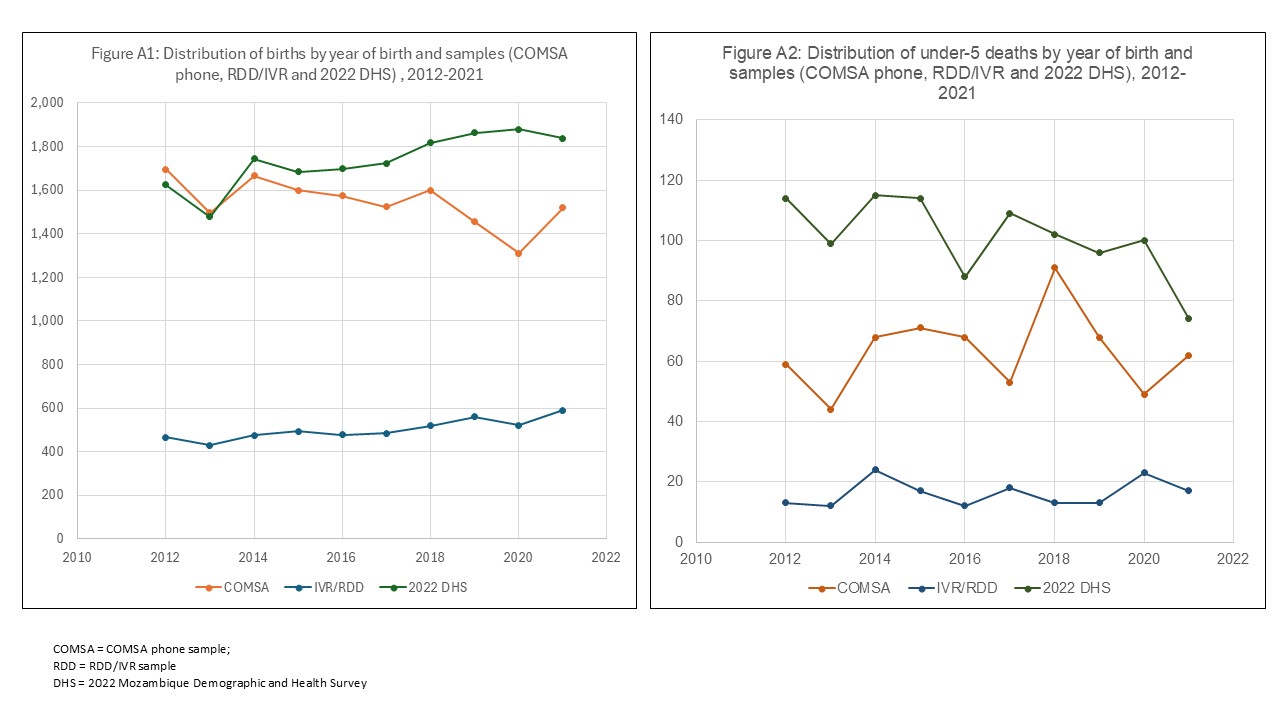

Supplement: Supplementary file 1 — Data S1. Supporting Information. [file TMI-30-1087-s001.zip › Fig A1-A2.JPG]

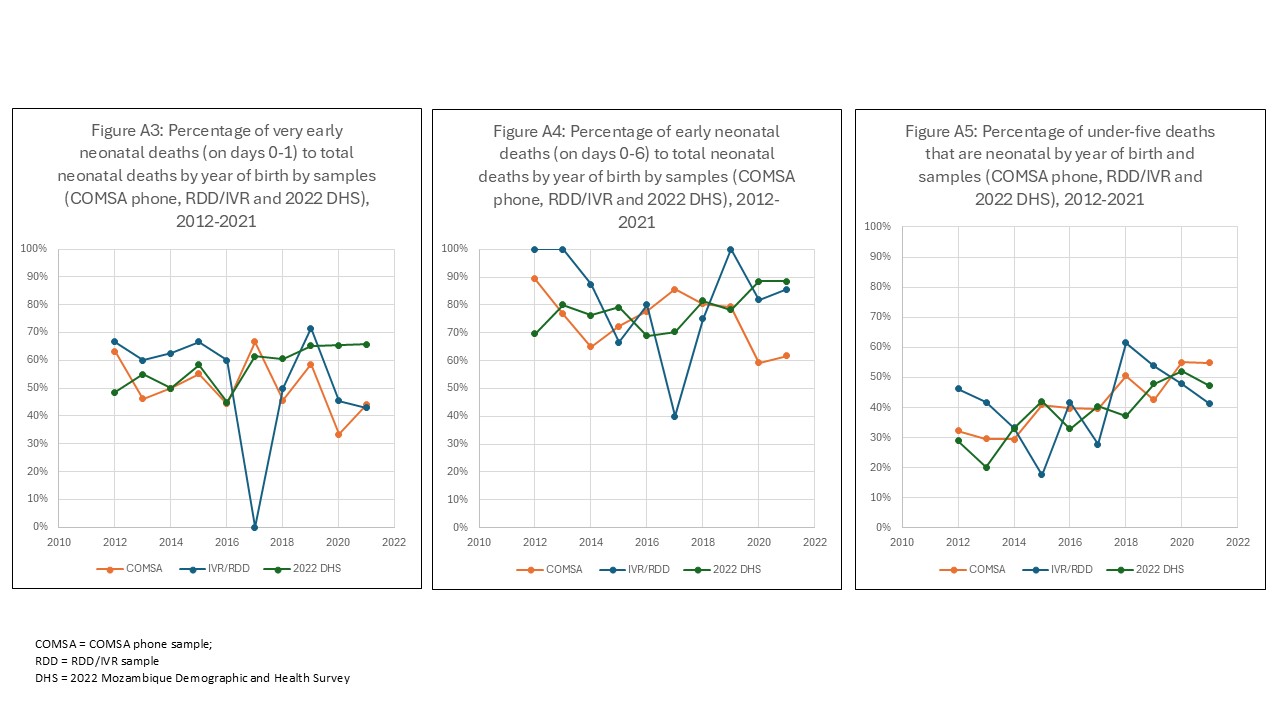

Supplement: Supplementary file 1 — Data S1. Supporting Information. [file TMI-30-1087-s001.zip › Fig A3-A4-A5.JPG]

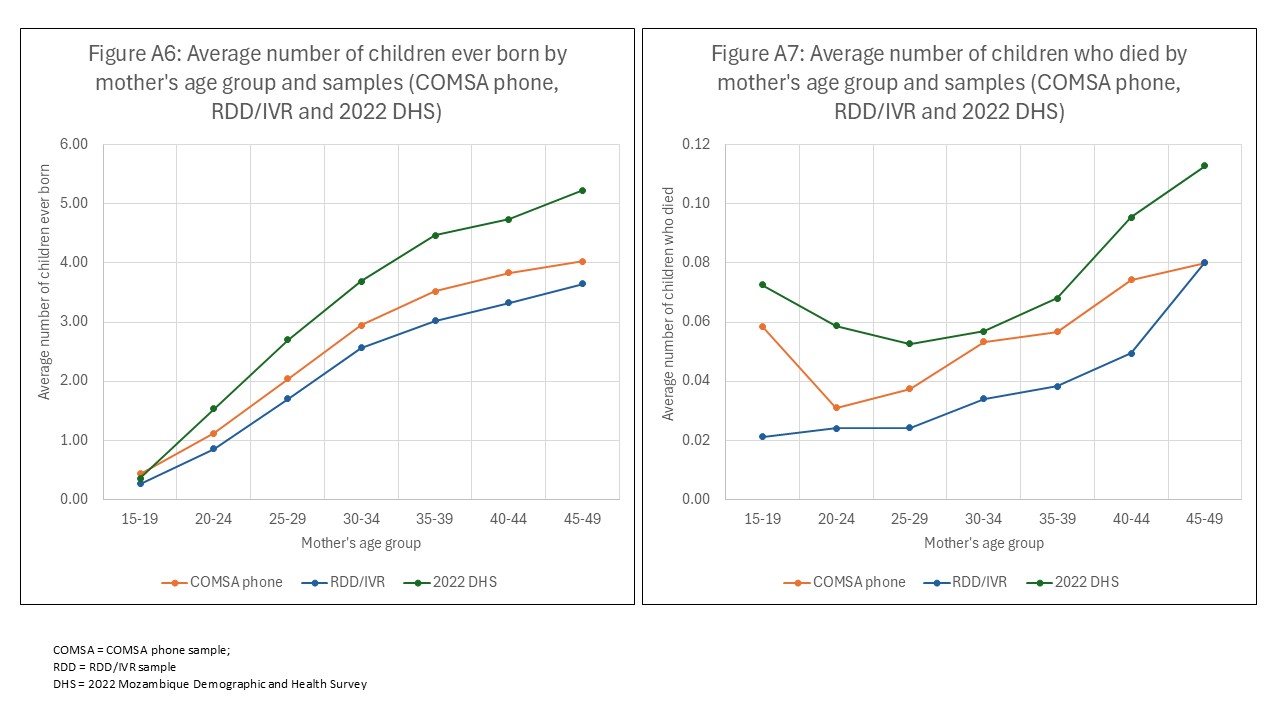

Supplement: Supplementary file 1 — Data S1. Supporting Information. [file TMI-30-1087-s001.zip › Fig A6-A7.jpg]
